# Using structural equation modelling to examine the relationship between place identity, sense of community, and environmental attitude

**DOI:** 10.1016/j.mex.2023.102443

**Published:** 2023-10-15

**Authors:** Elif Kutay Karaçor, Ezgi Akçam

**Affiliations:** a Landscape Architecture Department, Istanbul Technical University, Istanbul, Turkey; b Landscape Architecture Department, Duzce University, Duzce, Turkey

**Keywords:** Place identity, Sense of community, Environmental attitude, Structural equation modeling, Structural Equation Modeling

## Abstract

This study aimed to examine the association between place identity, sense of community, and environmental attitude. Within the theoretical framework, a connection has been identified among the variables of place identity, sense of community, and environmental attitude. However, the experimental analysis of this connection remains limited, with just a few research providing an explanation for the relationship between these three concepts. In this context, 121 inhabitants of Kültür neighborhood (Duzce City) were interviewed verbally. Structural equation modeling (SEM) was used to learn the relationship between these concepts.SEM is a statistical technique utilized in the social sciences to determine relationships between latent variables. To achieve this, oral interview data went through a reliability test using Statistical Package for the Social Sciences SPSS. Subsequently, confirmatory and regression analyses were conducted using LISREL to modify the model. Ultimately, the fit indices of the proposed model were assessed within the allowed range of fit values. The findings are significant in that they empirically highlight the importance of developing environmental and social policies to strengthen place identity and sense of community in order to improve environmental attitudes. As a result, it has been found that developing environmental awareness and consciousness is achievable through the bond and meaning that the community creates both within itself and with its place.

• The theoretical relationship presented in the literature was evaluated experimentally using this method.
• Data from oral interviews were analyzed using SPSS and LISREL softwares.

## Graphical abstract

**Figure:**
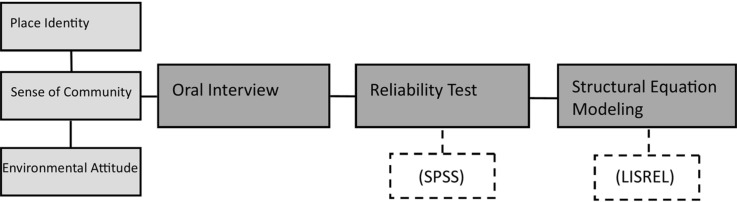

Specifications table

**Table:** 

Subject area:	Psychology
More specific subject area:	Place identity, Sense of community, Environmental attitude
Name of your method:	Structural Equation Modeling
Name and reference of original method:	Duncan, O. D. (1975). Introduction to Structural Equation Models. New York: Academic Press. ISBN 0–12–224,150–9.
Resource availability:	Not applicable

## Method details

Duncan [1] created structural equation modeling (SEM), a technique for analyzing the structural relationship among latent variables which is especially useful in the social sciences. This technique consists of a combination of factor analysis and multiple regression analysis. The purpose of using this technique is to measure and evaluate variables that can not be measured directly through observed variables. The hypothesis of this study is that "individuals' place identity, sense of community and environmental attitudes are effective on each other", and place identity, sense of community and environmental attitude are defined as latent variables. In order to test the hypothesis of this study, first of all, a scale related to place identity, sense of community and environmental attitude was developed (Table 1) and previous studies were used in the development of these scales [2], [3], [4], [5]. A one-page questionnaire comprised scale items created on a 5-point Likert scale (strongly disagree=1, disagree=2, undecided=3, agree=4, strongly agree=5).

**Table 1:** Place identity, sense of community and environmental attitude scale items.

Place Identity Scale Items	
V1	I feel like this neighborhood is a part of me
V2	I am very attached to this neighborhood
V3	This neighborhood means a lot to me
V4	I prefer this neighborhood to other neighborhoods
V5	There are places in this neighborhood that I'm emotionally attached to
V6	It's very difficult for me to leave this neighborhood
V7	Most of my life is spent around this neighborhood
V8	I would like to participate in events held in this neighborhood
V9	I'm proud of this neighborhood
V10	This neighborhood is the most ideal neighborhood I can live in.
V11	I identify strongly with this neighborhood
Sense of Community Scale Items	
V12	There is a close relationship among the people living in this neighborhood
V13	If I want to talk, I can find someone in this neighborhood to chat with
V14	I have similar characteristics to most of the people who live here
V15	People living in this neighborhood know that their neighbors will help them when they need help
V16	My friends in this neighborhood are also a part of my daily life
V17	If there is a problem in this neighborhood, the locals cooperate to solve that problem
V18	If someone does something good for this neighborhood, it makes me feel good too
V19	If I encounter an emergency/dangerous situation, even people I don't know in this neighborhood come to my aid
V20	The people living in this neighborhood take care of the poor and needy people of the neighborhood
V21	In this neighborhood, people greet
V22	Neighborhood relations have developed in this neighborhood
Environmental Attitude Scale Items	
V23	I research how to solve environmental problems
V24	I talk to other people about environmental issues
V25	I also try to raise awareness of my neighbors about environmental problems.
V26	I talk to my family about environmental issues
V27	I contribute to non-governmental organizations for environmental cleaning
V28	I use renewable energy sources
V29	I pay attention to water and electricity savings in housework

There are various approaches according to the tests and analysis techniques to be made regarding the sample size. Although it is suggested that the sample size should be sensitive to the number of items in the scale at a ratio of 5:1, or preferably 10:1, some researchers argue that at least 100 samples can be considered sufficient for latent variables in structural equation modeling [6], some researchers stated that a sample size of over 200 should be preferred [7]. In this study, it was aimed to interview 200 individuals in order to minimize the margin of error, but only 126 people aged 15 and over could be reached through simple random sampling. In determining the sample group as 15 years and older, especially the development of environmental attitude and sense of society after adolescence and the desire to take into account the views of this age group were effective. The total number of valid questionnaires accepted and transmitted to the SPSS database is 121.

Each scale transferred to SPSS was regarded as a single-factor structure, and exploratory factor analysis and reliability analysis were carried out on each of these scales. Thus, the reliability level of each scale and the level of explanation of the factor structures of the items in the scales were determined. Cronbach's Alpha (α) reliability levels were found to be 89.9% for the place identity scale, 88.2% for the sense of community scale, and 88.5% for the environmental attitude scale. In the exploratory factor analysis, it was determined that the factor loads of 11 items developed for the place identity, 11 items developed for the sense of community, and 7 items developed for the environmental attitude scales exceeded the acceptance value of 0.32 and were suitable for scale development. The explained variance was 50.56% for the place identity scale, 46.79% for the sense of community, and 59.59% for the environmental attitude. Considering that the variance explained in the single-factor designs is over 30% and the high reliability of the scales, the data analysis was transferred to the LISREL software and the structural equation model was applied.

In structural equation models, latent structures are examined through observed variables and thus the hypothesis established on latent variables is tested. According to the designed model, model parameters are calculated from the available data and iterative methods similar to factor analysis are used in this calculation process. In order to evaluate the model, various fit indices specified in the statistical literature are examined [8]. The criteria of these fit indices in the structural equation models and the cut-off points for acceptance are given in Table 2.

**Table 2:** Criteria of fit indices and cut-off points in the structural equation model [8].

Fit indices	Criteria	Cut-off points
χ2	*p*>0.05	–
χ2/sd	–	≤3=perfect ≤5=good
GFI/AGFI	0 (no fit) 1 (perfect fit)	≥0.90=good ≥0.95=perfect
RMSEA	0 (perfect fit) 1 (no fit)	≤0.05= perfect ≤0.08=good ≤0.10=acceptable
RMR/SRMR	0 (perfect fit) 1 (no fit)	≤0.05= perfect ≤0.08=good ≤0.10= acceptable
CFI	0 (no fit) 1 (perfect fit)	≥0.90=good ≥0.95= perfect
NFI/NNFI	0 (no fit) 1 (perfect fit)	≥0.90=good

Within the scope of this study, place identity, sense of community and environmental attitude were accepted as latent variables in order to test the hypothesis by creating a structural equation model based on the factors obtained by exploratory factor analysis, and the items assumed to be correlated by the latent variables were assigned as observed variables.

**Fig. 1:**
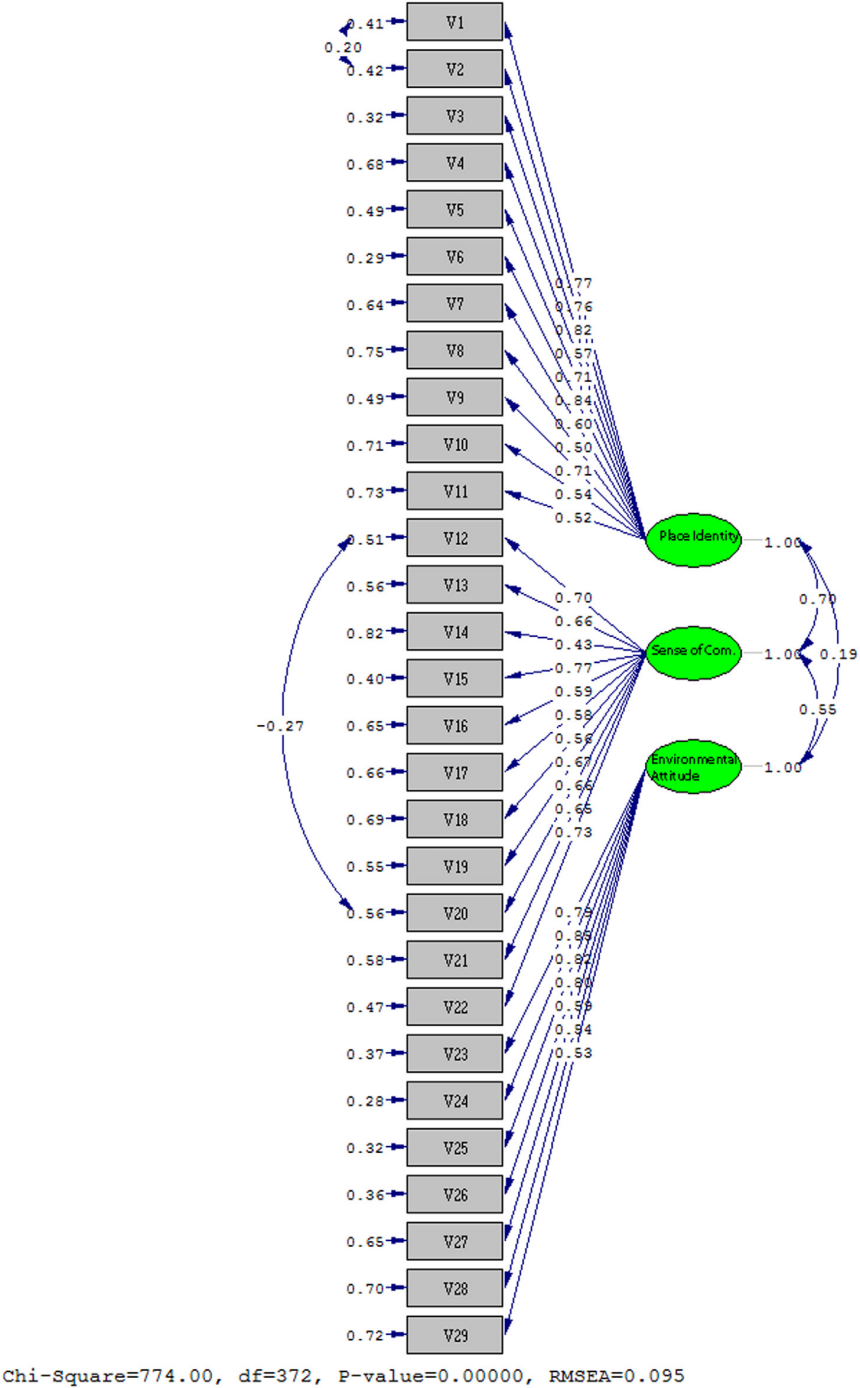
Path diagram of the model.

According to the analyzes made in line with the proposed model, firstly the significance level of the t value was checked and the explanations of the latent variables to the observed variables were checked. In this context, it was observed that all scale items gave a significant t value. However, when the modification indices that emerged as a result of the analyzes, it was determined that the modifications to be made between the items V1-V2 and V12-V20, respectively. Considering that the proposed modifications would not conflict with the theoretical structure, the iteration was continued in the analysis and the path diagram shown in Fig. 1 was created. The findings regarding the fit of the models are given in Table 3. When the results of the fit criteria of the proposed model were compared with the standard values, it was found that the results of the model were within acceptable fit values.

**Table 3:** Fit indices of the model.

Fit indices	1. Model	2. Model (V1-V2 Modification)	3. Model (V1-V2 and V12 –V20 Modification)
χ2/sd	837,19/374=2,24	814,80/373=2,18	774/372=2,08
RMSEA	0,10	0,099	0,095
RMR	0,12	0,12	0,12
SRMR	0,097	0,095	0,095
NFI	0,86	0,87	0,87
NNFI	0,91	0,92	0,92
CFI	0,92	0,92	0,93
GFI	0,68	0,68	0,69
AGFI	0,62	0,63	0,64

The study revealed that the correlation coefficient, which represents the standardized parameter value, between place identity and sense of community was determined as 0.70. Similarly, the correlation coefficient between place identity and environmental attitude variables was measured as 0.19. Additionally, the correlation coefficient between sense of community and environmental attitude variables was found as 0.55 (Fig. 1). These coefficients are positive and statistically significant at the 5% significance level. Based on these coefficients; The hypothesis that “individuals' place identity, sense of community and environmental attitudes are effective on each other” has been confirmed.

## Ethics statements

Informed consent was obtained from participants data has been fully anonymized.

## CRediT authorship contribution statement

**Elif Kutay Karaçor:** Supervision, Conceptualization, Methodology, Formal analysis, Writing – review & editing. **Ezgi Akçam:** Investigation, Methodology, Formal analysis, Writing – original draft.

## Declaration of Competing Interest

The authors declare that they have no known competing financial interests or personal relationships that could have appeared to influence the work reported in this paper

## Data Availability

Data will be made available on request.
